# Structural and Functional Characterization of a Novel Recombinant Antimicrobial Peptide from *Hermetia illucens*

**DOI:** 10.3390/cimb44010001

**Published:** 2021-12-21

**Authors:** Angela Di Somma, Antonio Moretta, Carolina Cané, Carmen Scieuzo, Rosanna Salvia, Patrizia Falabella, Angela Duilio

**Affiliations:** 1 Department of Chemical Sciences, University of Naples “Federico II”, Via Cinthia 4, 80126 Napoli, Italy; angela.disomma@unina.it (A.D.S.); carolina.cane@unina.it (C.C.); 2 National Institute of Biostructures and Biosystems (INBB), Viale Medaglie d’Oro 305, 00136 Roma, Italy; 3 Department of Sciences, University of Basilicata, 85100 Potenza, Italy; anto.moretta2711@gmail.com (A.M.); carmen.scieuzo@unibas.it (C.S.); r.salvia@unibas.it (R.S.); 4 Spinoff XFlies s.r.l., University of Basilicata, Via dell’Ateneo Lucano 10, 85100 Potenza, Italy; 5 CEINGE Biotecnologie Avanzate, 80145 Naples, Italy

**Keywords:** antimicrobial peptides, defensins, insects, *Hermetia illucens*, antibacterial activity

## Abstract

Antibiotics are commonly used to treat pathogenic bacteria, but their prolonged use contributes to the development and spread of drug-resistant microorganisms raising the challenge to find new alternative drugs. Antimicrobial peptides (AMPs) are small/medium molecules ranging 10–60 residues synthesized by all living organisms and playing important roles in the defense systems. These features, together with the inability of microorganisms to develop resistance against the majority of AMPs, suggest that these molecules might represent effective alternatives to classical antibiotics. Because of their high biodiversity, with over one million described species, and their ability to live in hostile environments, insects represent the largest source of these molecules. However, production of insect AMPs in native forms is challenging. In this work we investigate a defensin-like antimicrobial peptide identified in the *Hermetia illucens* insect through a combination of transcriptomics and bioinformatics approaches. The C-15867 AMP was produced by recombinant DNA technology as a glutathione S-transferase (GST) fusion peptide and purified by affinity chromatography. The free peptide was then obtained by thrombin proteolysis and structurally characterized by mass spectrometry and circular dichroism analyses. The antibacterial activity of the C-15867 peptide was evaluated in vivo by determination of the minimum inhibitory concentration (MIC). Finally, crystal violet assays and SEM analyses suggested disruption of the cell membrane architecture and pore formation with leaking of cytosolic material.

## 1. Introduction

The high increase in multidrug-resistant microorganisms is raising the challenge to find new molecules as an alternative to current antibiotics. Since the discovery of the first groups of antimicrobial peptides (AMPs), the magainins by Zasloff et al. [1,2,3], and the first AMP identified in the insect *Hyalophora cecropia* by Boman [4], a huge number of peptides have been found and studied. AMPs are amphiphatic small to medium size molecules consisting of 10 to 60 residues [5] produced by all living organisms and playing important roles in the defense systems [6,7]. Cationic peptides with a positive net charge due to the presence of basic residues (lysine and arginine) are the most widespread in nature [8].

For their high biodiversity, with over one million described species, insects represent the largest class of organisms, with a high ability to adapt to environmental changes and high resistance to a broad spectrum of pathogens [9,10]. Insects, lacking an adaptive immune system, base their defense/survival on the production of a multitude of broad-spectrum AMPs [11]; therefore, these organisms represent an essential source of biologically active compounds [12,13,14]. In particular, the dipteran *Hermetia illucens*, is considered an excellent model system for the identification and characterization of new AMPs since it is able to live in hostile environments rich in pathogens. *H. illucens* is a scavenger insect, well known for its ability to bioconvert decaying substrates [15] and considered then of scientific and economic interest in bioconversion processes, in animal feed and as an innovative and sustainable source of chitin, chitosan and lipids [16,17,18].

Several reports have shown that insect-derived AMPs are good candidates as alternatives to conventional antibiotics [19,20]. Most insect AMPs have a positive net charge that allows the interaction with the negatively charged molecules placed on the bacterial cell surfaces, such as the LPS of Gram-negative bacteria and teichoic acids of Gram-positive bacteria, respectively. Thus, electrostatic attraction is the first interaction occurring between peptides and cell membranes [21,22]. Then, the AMPs can exert their antibacterial activity through either membranolytic or non-membranolytic mechanisms [23]. Three putative models have been proposed so far to describe the membranolytic mechanism: the carpet model, in which AMPs can lead to bacterial death by membrane disintegration caused by micelle formation [24,25], the toroidal and the barrel-stave models, in which the AMP leads to bacterial death by pore formation into the bacterial outer membrane [26]. In the non-membranolytic mode of action, AMPs cause bacterial cell death by penetrating the outer membrane and interacting with intracellular targets involved in different biochemical pathways [27,28].

Based on their amino acid sequences and structures, insect AMPs can be classified in four groups: cysteine-rich peptides (e.g., defensins), α-helical peptides (e.g., cecropins), proline-rich peptides (e.g., drosocins) and glycine (Gly)-rich proteins (e.g., attacins) [11]. Defensins are small cationic peptides consisting of 34–51 residues with six conserved cysteines involved in the formation of three disulfide bridges according to the scheme Cys1-Cys4, Cys2-Cys5 and Cys3-Cys6 [29]. From a structural point of view, defensins show a *N*-terminal loop and an α-helix region followed by an antiparallel β-sheet [30,31]. Several insect defensins showed antibacterial activity against both Gram-positive and Gram-negative bacteria [32,33], including Defensin B from *Anomala cuprea* [34], the defensin peptide isolated from *Pyrrhocoris apterus* [35], Coprisin from *Copris tripartitus* [36], Defensin 1 isolated from *Acalolepta luxuriosa* [37] and the Defensin from *Bombus pascuorum* [38]. Insect defensins may kill bacteria through a membranolytic mechanism leading to pore formation on the bacterial membranes or could interact with phospholipids inducing microheterogeneity in the membrane [39,40]. However, recent reports suggest the occurrence of alternative mechanisms involving specific intracellular targets [41,42].

In this work we investigated a α-defensin-like antimicrobial peptide (C-15867 contig) identified in *Hermetia illucens* insect through a combination of transcriptomics and bioinformatics approaches [43]. The C-15867 α-defensin was produced by recombinant DNA technology and structurally and functionally characterized. The AMP was cloned and expressed in *Escherichia coli* as a glutathione S-transferase (GST) fusion peptide and purified by affinity chromatography. The free peptide was then obtained by thrombin proteolysis and structurally characterized by mass spectrometry and circular dichroism. Finally, the antibacterial activity of the C-15867 peptide was evaluated in vivo by determination of the minimum inhibitory concentration (MIC) on different bacterial strains and crystal violet assays and SEM analyses using *E. coli BL*_21_ as model.

## 2. Results

### 2.1. C-15867 ab Initio Modeling

**Figure 1:**
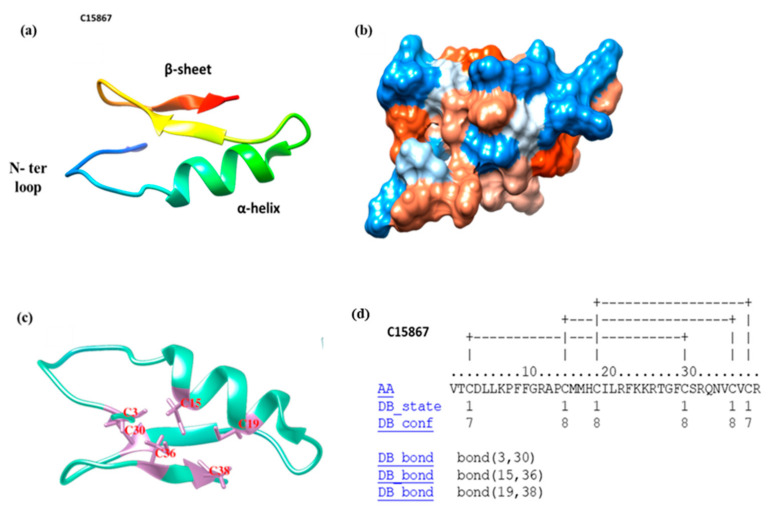
(**a**) C-15867 ribbon model: the N-ter loop, the α-helix region and the C-ter β-sheet are shown in different colors; (**b**) hydrophobicity surface of the C-15867 peptide. Hydrophobic residues are in orange-red while hydrophilic residues are highlighted in blue. The figure was generated with UCSF CHIMERA software [45]; (**c**) Cys3, Cys15, Cys19, Cys30, Cys36, Cys38 residues present in C-15867 peptide; (**d**) disulfide bonds pattern of C15867 predicted through the DISULFIND server. The figure was generated with UCSF CHIMERA software [45].

The C-15867 peptide sequence, VTCDLLKPFFGRAPCMMHCILRFKKRTGFCSRQNVCVCR, was identified in the *Hermetia illucens* insect through a combination of transcriptomics and bioinformatics approaches [43]. This sequence was analyzed with the BLAST software (https://blast.ncbi.nlm.nih.gov/Blast.cgi) accessed on 26 November 2021 showing a similarity with other antimicrobial peptides belonging to the defensins family sharing more than 50% homology with Defensin-like Peptide 4 (DLP4) from *H. illucens*, Aedes defensin A and B (AaeDefA, AeeDefB)) from *Aedes Aegypti* and Coprisin from *Copris tripartitu* (Supplementary Figure S1). A putative model of its three-dimensional structure was obtained using the I-TASSER web server and is shown in Figure 1a,b. The following values for the predicted structure were calculated: C-score = 0.14, TM-score = 0.73 ± 0.11, and RMSD = 1.9 ± 1.6 Å. The C-15867 peptide contains 6 Cys residues (Cys3, Cys15, Cys19, Cys30, Cys36, Cys38) as shown in Figure 1c. Pairing of the cysteine residues to form the correct scheme of disulfide bridges was predicted by the DISULFIND server and is reported in Figure 1d. The predicted scheme of connectivities, Cys1-Cys4, Cys2-Cys5 and Cys3-Cys6, corresponds to the classical arrangement of the C6 class of the cis-defensin superfamily and the insect class I defensins [44].

### 2.2. Expression, Purification and Characterization of the Recombinant GST-Peptide Fusion Product

**Figure 2:**
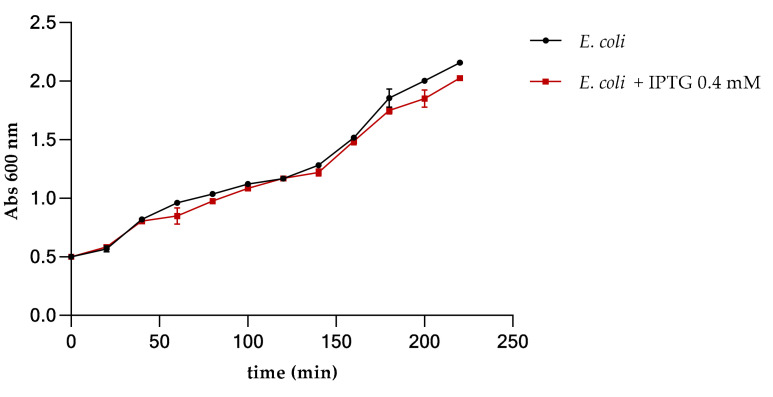
Growth curve of *E. coli BL*_21_ in the presence and in the absence of IPTG 0.4 mM for the over expression of GST-peptide. The error bars on the graphs stand for the standard deviation from the mean of three experiments.

The recombinant GST peptide fusion product was expressed in *E. coli BL*_21_. The possible toxicity of the peptide during the overexpression was evaluated by monitoring the bacterial growth with and without IPTG induction. Figure 2 shows that no significant effects on bacterial growth occurred following induction and GST-peptide production.

**Figure 3:**
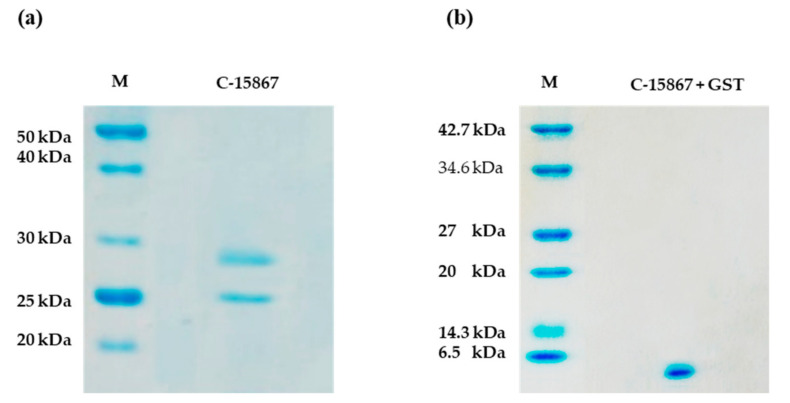
(**a**) SDS-PAGE of the purified recombinant GST-peptide fusion product (2 µg). The band at about 29 kDa corresponds to the expected product while the band at about 25 kDa represents free GST. The amount of GST-peptide fusion product is equal to 3.2 mg per liter of culture. (**b**) SDS-PAGE (12.5%) of recombinant C-15867 after cleavage with thrombin (2 µg). The peptide yield is 1.8 mg per liter of culture.

The GST-peptide was purified by affinity chromatography using a GSTrap FF column equilibrated with binding buffer. After several washes the GST-fused peptide was eluted with an excess of reduced glutathione. The purity of the recombinant GST-peptide fusion product was evaluated by SDS-PAGE (Figure 3) and its primary structure verified by MALDI mass spectrometry (Supplementary Figure S2).

Figure 3a shows the resulting SDS-PAGE analysis exhibiting the expected peptide band at 29 kDa corresponding to the molecular weight of the recombinant GST-fused peptide together with a less intense band at 25 kDa corresponding to native GST. The presence of the GST band may be due to intracellular cleavage of the fusion product or to translational pausing at the junction between GST and the fusion partner. The recombinant GST-C-15867 band was excised from the gel, digested with trypsin and the resulting peptide mixture directly analyzed by MALDI-MS/MS tandem mass spectrometry. The mass signals recorded in the spectra were assigned to the anticipated GST-peptide sequence on the basis of their molecular mass and the fragmentation spectrum confirming the correctness of the recombinant product. (Supplementary Figure S2).

### 2.3. Isolation and Characterization of Free C-15867 Peptide

The recombinant C-15867 peptide was released from the GST by enzymatic digestion with thrombin. The GST-C-15867 fused peptide was trapped onto glutathione-conjugated agarose beads and incubated with thrombin directly on the resin. Following thrombin digestion, the native peptide was released while the GST protein was retained on the beads. The purity of peptide was assessed by SDS-PAGE showing the occurrence of a single band corresponding to the expected electrophoretic mobility at about 4.5 kDa (Figure 3b). The C-15867 band was excised from the gel, digested with trypsin and the resulting peptide mixture directly analyzed by MALDI-MS/MS tandem mass spectrometry. The mass signals recorded in the spectra were assigned to the anticipated C-15857 sequence on the basis of their molecular mass and the fragmentation spectrum confirming the structure of the antimicrobial peptide. Table 1 reports the assignments of the mass signals recorded in the spectra to the corresponding peptides.

### 2.4. Circular Dichroism Analyses

**Figure 4:**
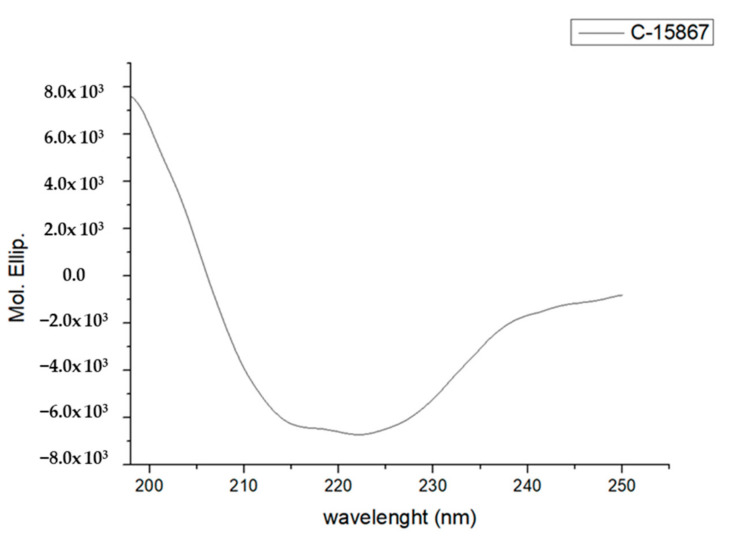
CD spectrum of the native C-15867 peptide in PBS buffer.

Circular dichroism analysis was carried out to verify the secondary structure elements of the native peptide. Figure 4 reports the corresponding CD spectrum recorded in PBS buffer displaying a pronounced minimum at about 215 nm and a maximum at 202 nm typical of the β-sheet structure. The second minimum around 222 nm demonstrated the presence of the α-helix structure. Deconvolution of the CD spectrum revealed the occurrence of 36.2% β sheet structure, 7.5% α-helix and 11.5% turn according to the general secondary structure of the defensin family.

### 2.5. Antimicrobial Activity Assays

**Figure 5:**
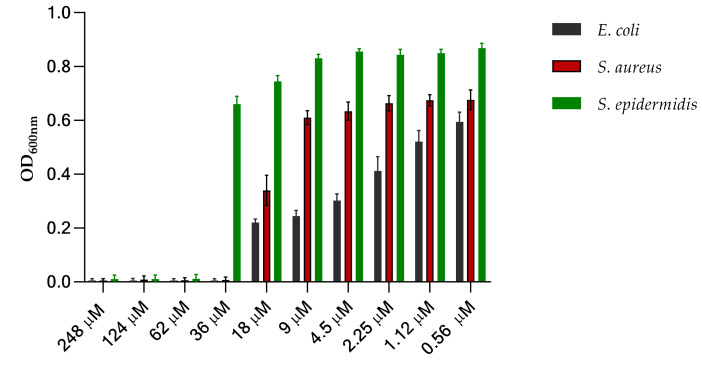
Determination of C-15867 minimum inhibitory concentration (MIC). All bacteria cells were grown in the presence of serial dilution of peptide from 248 µM to 0.56 µM and incubated at 37 °C for 24 h. The error bars on the graphs stand for the standard deviation from the mean of three experiments.

The *in vivo* antimicrobial activity of the recombinant C-15867 peptide was evaluated against three different bacterial strains: *E. coli BL*_21_, *Staphylococcus aureus NCTC 12493* and *Staphylococcus. epidermidis ATCC 12228*. The minimum inhibitory concentration (MIC) was determined as the lowest concentration showing no visible growth after 24 h of incubation at 37 °C. The obtained results are illustrated in Figure 5 demonstrating that the peptide exerted a strong antimicrobial effect on all bacterial strains. In particular, the calculated minimum inhibitory concentration (MIC) on *E. coli BL*_21_ and *S. aureus* cells was 36 μM.

The antimicrobial activity of defensin and defensin-like peptides is strictly related to their three-dimensional structure involving three disulphide bridges. The C-15867 peptide was incubated in the presence of 3 mM DTT for 1 h at 56 °C to reduce the S-S bridges and the antimicrobial activity of the reduced peptide was evaluated. Supplementary Figure S3 showed that bacterial growth was not affected by different concentrations of the reduced peptide demonstrating that reduction of S-S bridges completely abolished the antibacterial activity of the C-15867 peptide.

**Figure 6:**
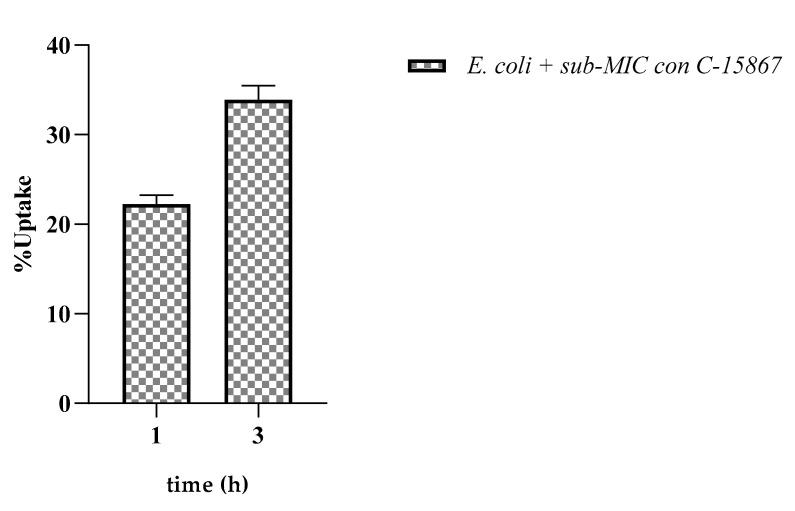
Effect of C-15867 on *E. coli BL*_21_ membrane permeability. The uptake of crystal violet was calculated in the presence of sub-mic concentration (9 µM) of the peptide.

With C-15867 being a defensin-like AMP, we investigated the effect of the peptide, if any, on *E. coli BL*_21_ membrane permeability using the crystal violet assay. Bacterial cells were grown in the presence of a sub-mic concentration (9 µM) of the peptide and the uptake of crystal violet was monitored at different times of incubation. Figure 6 shows a 22% and 34% increase in the uptake of crystal violet following 1 h and 3 h of treatment, respectively, suggesting that the peptide induced an alteration in the permeability of the *E. coli BL*_21_ cell membrane.

### 2.6. Scanning Electron Microscopy (SEM) Analyses

The effect of C-15867 on the morphology of *E. coli BL*_21_ cells was also assessed by SEM microscopy analyses. Bacterial cells were treated with a sub-mic concentration (9 µM) of the antimicrobial peptide and SEM analyses were carried out after 3 h of incubation in comparison with untreated cells.

**Figure 7:**
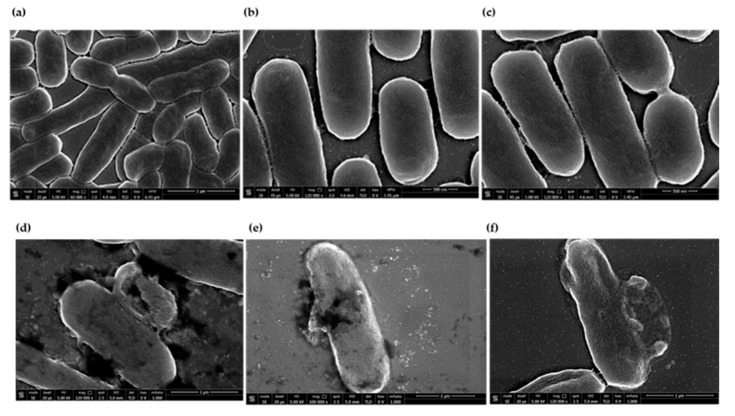
Scanning electron microscopy of *E. coli BL_2_*_1_ cells following 3 h treatment with a sub-mic concentration (9 µM) of the antimicrobial peptide C-15867 (**d**–**f**) in comparison with untreated cells (**a**–**c**). The scale bar was 2 µm in panel (**a**), 500nm in panel (**b**,**c**) and 1µm in panel (**d**–**f**).

Figure 7 shows that untreated cells were normal in shape with smooth surfaces (Figure 7a–c) whereas those treated with the C-15867 peptide clearly exhibited severe morphological changes (Figure 7d–f). The cell wall and the cell surface were rough gradually forming pores with subsequent leakage of cytoplasmic material confirming the disrupting effect of the antimicrobial peptide on the bacterial membrane.

## 3. Discussion

Antimicrobial peptides (AMPs) represent an essential class of effector molecules of the host innate immune system acting as the first line of defense against pathogen invasion. In addition to its action against microorganisms, AMPs are able to modulate the innate and adaptive immunity by inducing the expression of proinflammatory cytokines and chemokines, chemotaxis, apoptosis, and gene transcription. These effects, together with the inability of microorganisms to develop resistance against the majority of AMPs, indicated these molecules as effective alternatives to classical antibiotics opening up a new way for drug discovery and development [46].

Insects produce complex and diverse sets of chemicals for survival and defense and are among the richest sources of antimicrobial peptides due to their extensive biodiversity and their heterogeneous living environment [11,47]. The insect AMPs generally have a broad spectrum of activity, being effective against bacteria, fungi and also able to inhibit virus replication. Accordingly, in recent years, an increasing number of insect AMPs have been reported showing several applications in pharmaceutical, biomedical and agricultural fields [48]. The immune system of the black soldier fly *H. illucens* is highly developed as it feeds on decaying substrates [15] and displays a supernormal capacity to survive in hostile environments rich in pathogenic microorganisms. Therefore, *H. illucens* might represent one of the most promising sources for identification of new AMPs. However, discovery, production, and functional investigation of AMPs from *H. illucens* still remain at a preliminary stage [49]. Recently, putative AMPs sequences have been identified in *H. illucens* through a combination of transcriptomics and bioinformatics approaches [43]. These features were used to select the C-15867 peptide as an excellent putative candidate to be produced and characterized. A putative model of its three-dimensional structure obtained by docking calculation predicted a tertiary structure very similar to the defensin family. Accordingly, the C-15867 peptide sequence contains six Cys residues, a common characteristic of the insect defensins, involved in the formation of three disulfide bonds that are predicted to be arranged as Cys1-Cys4, Cys2-Cys5, Cys3-Cys6 [40]. The presence of lysine and arginine residues confers a positive net charge to the peptide, which is essential for the interaction with the negatively charged molecules placed on the bacterial membranes.

In order to explore the biological properties of insect AMPs, several different approaches have been developed over the years for the production of AMPs including chemical synthesis or recombinant expression. However, any attempt to produce C-15687 by chemical methods resulted in a peptide devoid of antimicrobial activity most likely because of the lack of the correct pattern of disulfide bridges. We then pointed out the molecular biology techniques which were considered the most effective method to obtain AMPs with the native structure and the correct folding [50] using heterologous expression in *E. coli* cells.

However, AMPs are small molecules toxic to the bacterial host cells and cannot be expressed directly in the cells [51]. We therefore expressed the C-15867 peptide in the *E. coli* cells as a GST-fused peptide to decrease the lethal effect of the peptide towards the host system [52]. Antimicrobial assays performed before and after IPTG induction demonstrated that the GST-fused peptide did not show any toxicity on the bacterial cells allowing a correct growth of *E. coli BL*_21_ and the correct expression of the recombinant product.

Very likely the presence of the GST protein tag impaired the antibacterial activity of the peptide. Moreover, the presence of GST was effective in (i) driving the correct folding of the peptide as demonstrated by functional experiments (see below) [53] and (ii) allowing purification of the recombinant product by a simple and straightforward procedure. The recombinant GST-fused peptide could indeed be easily purified by a single step of GST affinity chromatography using glutathione-conjugated agarose beads leading to a homogeneous product as demonstrated by SDS PAGE and mass spectrometry analyses. The C-15867 peptide was eventually released from the GST fusion product by enzymatic digestion with thrombin to originate the free form of the peptide that could be structurally and functionally characterized.

The primary structure of the peptide was assessed by MALDI mass mapping resulting in the verification of its complete amino acid sequence. The secondary structure of C-15867 was determined by circular dichroism spectroscopy showing the expected occurrence of both α-helix and β-sheet elements for a member of the defensin family, confirming docking predictions.

Finally, the C-15867 peptide showed good antimicrobial activity against *E. coli*, *S. aureus* and *S. epidermidis* cells exhibiting a minimum inhibitory concentration in the low micromolar range. Zhao et al. [54] discussed a defensin peptide deriving from *Venerupis philippinarum* where the MIC was determined against different bacteria, which resulted in a low concentration range from 1.64 to 26.26 µM. The MIC values calculated for the C-15867 peptide, 36 µM on both *E. coli* and *S. aureus* are then in perfect agreement with the data reported in literature for other defensins.

Since C-15867 is a defensin-like AMP, we investigated the effect of the peptide on the *E. coli* membrane by crystal violet assays and SEM analyses. Peptide C-15867 was demonstrated to perturb the integrity of cell membrane allowing the uptake of the violet dye inside the cell. This effect was confirmed by SEM observations showing alteration of cell membrane architecture and pore formation with leaking of cytosolic material.

These results indicate that C-15867 has great potential for treating infections caused by antibiotic resistant bacteria and open up the way to the production of other AMPs from *H. illucens* identified by bioinformatic analyses using molecular biology techniques.

## 4. Materials and Methods

### 4.1. C-15867 ab Initio Molecular Modeling

The C-15867 peptide, previously identified in the *H. illucens* transcriptome [55] was analyzed in Moretta et al., 2020 [43], through bioinformatics strategies and functionally annotated as Defensin by the Blast2Go software. Because of its high prediction scores for the antimicrobial activity and its sequence length, it has been selected in this work to be *in vitro* studied. Thus, it was *ab initio* modelled through the I-TASSER webserver [56,57,58]. The generated model confidence is quantitatively measured by the C-score, which is calculated based on the significance of threading template alignments and the convergence parameters of the structure assembly simulations. C-score is typically in the range from −5 to +2, where a positive C-score value represents a model with a high confidence and vice-versa. TM-score and RMSD values are estimated based on C-score and protein length following the correlation observed between these qualities. Since the C-15867 peptide belongs to defensin AMP family, the conformation of the disulfide bonds was in silico evaluated through the DISULFIND webserver [59,60,61,62].

### 4.2. Molecular Cloning of the C-15867 Gene

To generate the C-15867 gene insert, cDNA was amplified by a polymerase chain reaction (PCR) and was performed using KOD DNA Polymerase (Merck Millipore, Burlington, MA, USA), according to the manufacturer’s protocol and the specific primers f containing the restriction sites for BamHI and EcoRI (underlined) were:

Hill_BB_C-15867FOR 5′ GGATCCGTCACCTGTGATCTTCTAAA 3′

Hill_BB_C-15867REV 5′GAATTCTTATCTGCACACGCAAACG 3′

The PCR product was checked on agarose (1.2% *w*/*v*) gel electrophoresis.

Once the C-15867 fragment was ligated in pCR™II-TOPO^®^ vector, the construct was then transformed into competent TOP10 cells. The identification of the transformed *E. coli* colony with the recombinant plasmid was confirmed with a miniprep procedure (FastPlasmid Mini Kit, 5 PRIME) and its subsequent hydrolysis with restriction enzymes. The construct was sequenced (Macrogen Europe, Amsterdam, The Netherlands) to ensure the peptide sequence was not affected by mutations. The plasmid DNA was then digested with EcoRI (20 U/μL) and BamHI (20 U/μL) and the fragment corresponding to the peptide gene was purified using Quantum Prep Freeze N-Squeeze DNA Gel Extraction Spin Columns (Bio-Rad, Hercules, CA, USA) according to the manufacturer’s protocol and used for the ligation reaction in pGEX-4T1 expression plasmid (Novagen, Madison, WI, USA). The ligation mixture was used to transform *E. coli* DH5α chemically competent cells and the positive screening was then conducted through a miniprep procedure and the sequence was verified by sequencing (Macrogen Europe, Amsterdam, The Netherlands) to ensure the corrected frame of the C-15867 with the GST sequence.

### 4.3. Recombinant Production of the GST-C-15867 Antimicrobial Peptide

Recombinant C-15867 was expressed in *E. coli* *BL*_21_ cells, in Luria Bertani medium at 37 °C supplemented with 100 µg/mL ampicillin in order to select the strain of interest. The expression of the fusion product was induced in the exponential phase with 0.4 mM of isopropyl-thio-β-*D*-galactoside (IPTG). After growth at 20 °C for 16 h, bacterial cells were harvested by centrifugation at 5000 rpm for 20 min at 4 °C, lysed by resuspending bacteria pellets in 0.01 M Na_2_HPO_4_, 0.15 M NaCl, 5mM DTT, pH 7.4, and then sonicated for 20 min. The lysate was incubated in static condition at 25 °C for 30 min with triton 1% and centrifuged at 15,000 rpm for 60 min at 4 °C, allowing the separation of the insoluble fraction from the soluble sample. The GST-peptide fusion product was then purified from the soluble fraction by affinity chromatography on GSTrap^TM^ FF 1 mL connected to a Fast Liquid Protein Chromatography system (AKTA plus). The column was equilibrated with 0.01 M Na_2_HPO_4_, 0.15 M NaCl, 5 mM DTT, pH 7.4 and eluted with 10 mM reduced glutathione in 25 mM Tris-HCl pH 8.0, 150 mM NaCl, 5 mM DTT. Peptide purity was verified by SDS-PAGE 12,5% and its primary structure was validated by MALDI mapping strategy on a 5800 MALDI-TOF/TOF instrument (ABI Sciex, Redwood City, CA, USA).

### 4.4. Cleavage of GST-Peptide Fusion Product

The cleavage of peptide from GST protein was carried out with thrombin from bovine plasma (Sigma–Aldrich, St. Louis, MO, USA) 6U for mg of peptide for 16–24 h at 4 °C. The cleaved peptide solution was applied to affinity chromatography to purify the active form of C-15867 with glutathione-agarose (Sigma). The peptide was separated by the thrombin using centrifugal filters (Sigma–Aldrich, St. Louis, MO, USA) cutoff 10 kDa. Protein concentration was determined with the Bradford Reagent (Sigma–Aldrich, St. Louis, MO, USA), using BSA as a standard.

### 4.5. Determination of the Minimum Inhibitory Concentration Values of C-15867 Peptide

The different strains (*E. coli BL*_21_, *Staphylococcus aureus NCTC 12493* and *Staphylococcus epidermidis ATCC 12228*) were grown in the presence of serial dilution of C-15867 from 248 μM to 0.5 μM and the MIC values were determined as the lowest concentration showing no visible growth after 24 h of incubation at 37 °C. The procedure was performed using medium as negative control and cells approximately 5 × 10^5^ CFU/mL as positive control to monitor the normal growth of bacteria.

Reduction of the S-S bridges was carried out by incubation of the peptide with 3 mM of DTT, pH 7.4 at 56 °C for 1 h. *E. coli Bl*_21_ cells were treated with different concentrations of the reduced C-15867 peptide from 0.56 to 298 µM and the cell growth was evaluated by measuring the OD_600_ after overnight incubation at 37 °C.

### 4.6. Circular Dichroism Analyses

The secondary structure of C-15867 was analyzed by circular dichroism (CD) spectra at 25 °C using a 1 cm quartz cell using a 198–250 nm measurement range, 20 nm/min scanning speed, 0.2 nm data pitch. Peptide concentration for CD measurement was 2 μM in PBS buffer, pH 7.4. The secondary structure was determined by BeStSel (Beta Structure Selection) software, a novel method for the secondary structure determination which fits the experimental CD curve by the linear combination of fixed basis components to get the proportion of the eight structural elements. The CD signal was transformed into molar ellipticity using Spectra Manager and calculating the concentration per residue of C-15867.

### 4.7. Crystal Violet Assays

The alteration in membrane permeability was detected by crystal violet assay [63]. Bacterial cells were grown in LB broth at 37 °C in the presence of 10 μM peptide for 1 and 3 h. Subsequently, the cells were harvested at 9300× *g* for 5 min and then the cells were resuspended in 1× PBS containing 10 g/mL of crystal violet. The cell suspension was incubated for 10 min at 37 °C and then centrifuged at 13,400× *g* for 15 min and the OD_590_ of the supernatant was measured at the spectrophotometer. Control samples were prepared similarly without treatment. The OD value of the crystal violet solution, which was originally used in the assay, was taken and it was considered as 100%. The percentage of crystal violet uptake of all the samples was quantified according to the following formula: OD value of the sample/OD value of the crystal violet solution 100×.

### 4.8. SEM Measurements

*E. coli BL*_21_ cells were grown in the absence and in the presence of native C15867 peptide at 37 °C for 3 h. Samples from each culture were fixed in 2.5% glutaraldehyde solution for 16 h. Following the incubation, the bacterial cells were washed with 0.1 M PBS (pH 7.2) and dehydrated in a series of ethanol solutions ranging from 30 to 100%, followed by three washes with 100% ethanol. *E. coli* cells were deposited onto glass substrate first sputter coated with a thin layer of Au-Pd alloy (Denton Vacuum Desk V Magnetron Sputtering) to allow subsequent morphological characterization using a FEI Nova NanoSEM 450.

## Figures and Tables

**Table 1:** Mass signals recorded in the MALDI-MS/MS spectra of the tryptic digest of carboxyamidomethylated C-15867 peptide. The corresponding peptides are reported.

Theoretical *m*/*z*	Experimental *m*/*z*	Peptide Sequence
727.25	727.32	TGFCSR
935.45	935.41	QNVCVCR
1320.57	1320.60	APCMMHCILR
1452.82	1452.82	VTCDLLKPFFGR
1723.83	1723.82	APCMMHCILRFKK
1297.92	1297.93	KRTGFCSR QNVCVCR

## Data Availability

Not applicable.

## Supplementary Materials

The following are available online at https://www.mdpi.com/article/10.3390/cimb44010001/s1.

## References

[1] Zasloff M. (1987). Magainins, a class of antimicrobial peptides from Xenopus skin: Isolation, characterization of two active forms, and partial cDNA sequence of a precursor. Proc. Natl. Acad. Sci. USA.

[2] Zasloff M. (2002). Antimicrobial peptides of multicellular organisms. Nature.

[3] Soravia E., Martini G., Zasloff M. (1988). Antimicrobial properties of peptides from Xenopus granular gland secretions. FEBS Lett..

[4] Steiner H., Hultmark D., Engström Å., Bennich H., Boman H.G. (1981). Sequence and specificity of two antibacterial proteins involved in insect immunity. Nature.

[5] Huan Y., Kong Q., Mou H., Yi H. (2020). Antimicrobial peptides: Classification, Design, Application and Research Progress in Multiple Fields. Front. Microb..

[6] Borah A., Deb B., Chakraborty S. (2020). A crosstalk on antimicrobial peptides. Int. J. Pept. Res. Ther..

[7] Mahlapuu M., Håkansson J., Ringstad L., Björn C. (2016). Antimicrobial peptides: An emerging category of therapeutic agents. Front. Cell. Infect. Microbiol..

[8] Malik E., Dennison S.R., Harris F., Phoenix D.A. (2016). pH dependent antimicrobial peptides and proteins, their mechanisms of action and potential as therapeutic agents. Pharmaceuticals.

[9] Aminov R.I. (2010). A brief history of the antibiotic era: Lessons learned and challenges for the future. Front. Microbiol..

[10] Wu Q., Patočka J., Kuča K. (2018). Insect antimicrobial peptides, a mini review. Toxins.

[11] Manniello M.D., Moretta A., Salvia R., Scieuzo C., Lucchetti D., Vogel H., Sgambato A., Falabella P. (2021). Insect antimicrobial peptides: Potential weapons to counteract the antibiotic resistance. Experientia.

[12] Scieuzo C., Salvia R., Franco A., Pezzi M., Cozzolino F., Chicca M., Scapoli C., Vogel H., Monti M., Ferracini C. (2021). An integrated transcriptomic and proteomic approach to identify the main *Torymus sinensis* venom components. Sci. Rep..

[13] Salvia R., Scieuzo C., Grimaldi A., Fanti P., Moretta A., Franco A., Varricchio P., Vinson S.B., Falabella P. (2021). Role of Ovarian Proteins Secreted by *Toxoneuron nigriceps* (Viereck) (Hymenoptera, Braconidae) in the Early Suppression of Host Immune Response. Insects.

[14] Salvia R., Grimaldi A., Girardello R., Scieuzo C., Scala A., Bufo S.A., Vogel H., Falabella P. (2019). *Aphidius ervi* teratocytes release Enolase and Fatty Acid Binding Protein through exosomal vesicles. Front. Physiol..

[15] Scala A., Cammack J.A., Salvia R., Scieuzo C., Franco A., Bufo S.A., Tomberlin J.K., Falabella P. (2020). Rearing substrate impacts growth and macronutrient composition of *Hermetia illucens* (L.) (Diptera: Stratiomyidae) larvae produced at an industrial scale. Sci. Rep..

[16] Salvia R., Falabella P. (2021). Bioconverter insects: A good example of circular economy, the study case of *hermetia illucens*. An Introduction to the Circular Economy.

[17] Triunfo M., Tafi E., Guarnieri A., Scieuzo C., Hahn T., Zibek S., Salvia R., Falabella P. (2021). Insect Chitin-Based Nanomaterials for Innovative Cosmetics and Cosmeceuticals. Cosmetics.

[18] Hahn T., Tafi E., Paul A., Salvia R., Falabella P., Zibek S. (2020). Current state of chitin purification and chitosan production from insects. J. Chem. Technol. Biotechnol..

[19] Hancock R.E.W., Sahl H.G. (2006). Antimicrobial and host-defense peptides as new anti-infective therapeutic strategies. Nat. Biotechnol..

[20] Narayana J.L., Chen J.-Y. (2015). Antimicrobial peptides: Possible anti-infective agents. Peptides.

[21] Bechinger B., Gorr S.U. (2017). Antimicrobial peptides: Mechanisms of action and resistance. J. Dent. Res..

[22] Brown K.L., Hancock R.E. (2006). Cationic host defense (antimicrobial) peptides. Curr. Opin. Immunol..

[23] Di Somma A., Moretta A., Canè C., Cirillo A., Duilio A. (2020). Antimicrobial and antibiofilm peptides. Biomolecules.

[24] Kumar P., Kizhakkedathu J.N., Straus S.K. (2018). Antimicrobial peptides: Diversity, mechanism of action and strategies to improve the activity and biocompatibility in vivo. Biomolecules.

[25] Goyal R.K., Mattoo A.K. (2016). Plant antimicrobial peptides. Host Defense Peptides and Their Potential as Therapeutic Agents.

[26] Travkova O.G., Moehwald H., Brezesinski G. (2017). The interaction of antimicrobial peptides with membranes. Adv. Colloid. Interface Sci..

[27] Lee H., Lim S.I., Shin S.H., Lim Y., Koh J.W., Yang S. (2019). Conjugation of cell-penetrating peptides to antimicrobial peptides enhances antibacterial activity. ACS Omega.

[28] Di Somma A., Avitabile C., Cirillo A., Moretta A., Merlino A., Paduano L., Duilio A., Romanelli A. (2020). The antimicrobial peptide Temporin L impairs *E. coli* cell division by interacting with FtsZ and the divisome complex. BBA-Gen. Subj..

[29] Bachère E., Destoumieux D., Bulet P. (2000). Penaeidins, antimicrobial peptides of shrimp: A comparison with other effectors of innate immunity. Aquaculture.

[30] Koehbach J. (2017). Structure-activity relationships of insect defensins. Front. Chem..

[31] Amerikova M., Pencheva El-Tibi I., Maslarska V., Bozhanov S., Tachkov K. (2019). Antimicrobial activity, mechanism of action, and methods for stabilisation of defensins as new therapeutic agents. Biotechnol. Biotechnol. Equip..

[32] Parisi K., Shafee T.M., Quimbar P., van der Weerden N.L., Bleackley M.R., Anderson M.A. (2019). The evolution, function and mechanisms of action for plant defensins. Seminars in Cell Developmental Biology.

[33] Tonk M., Knorr E., Cabezas-Cruz A., Valdés J.J., Kollewe C., Vilcinskas A. (2015). *Tribolium castaneum* defensins are primarily active against Gram-positive bacteria. J. Invertebr. Pathol..

[34] Yamauchi H. (2001). Two novel insect defensins from larvae of the cupreous chafer, *Anomala cuprea*: Purification, amino acid sequences and antibacterial activity. Insect Biochem. Mol. Biol..

[35] Cociancich S., Dupont A., Hegy G., Lanot R., Holder F., Hetru C., Hoffmann J.A., Bulet P. (1994). Novel inducible antibacterial peptides from a hemipteran insect, the sap-sucking bug *Pyrrhocoris apterus*. Biochem. J..

[36] Hwang J.-S., Lee J., Kim Y.-J., Bang H.-S., Yun E.-Y., Kim S.-R., Suh H.-J., Kang B.-R., Nam S.-H., Jeon J.-P. (2009). Isolation and Characterization of a Defensin-Like Peptide (Coprisin) from the Dung Beetle, *Copris tripartitus*. Int. J. Pept..

[37] Ueda K., Imamura M., Saito A., Sato R. (2005). Purification and cDNA cloning of an insect defensin from larvae of the longicorn beetle, *Acalolepta luxuriosa*. Appl. Entomol. Zool..

[38] Rees J.A., Moniatte M., Bulet P. (1997). Novel antibacterial peptides isolated from a European bumblebee, *Bombus pascuorum* (Hymenoptera, apoidea). Insect Biochem. Mol. Biol..

[39] Robles-Fort A., García-Robles I., Fernando W., Hoskin D.W., Rausell C., Real M.D. (2021). Dual antimicrobial and antiproliferative activity of TcPaSK peptide derived from a *Tribolium castaneum* insect defensin. Microorganisms.

[40] Sheehan G., Garvey A., Croke M., Kavanagh K. (2018). Innate humoral immune defences in mammals and insects: The same, with differences?. Virulence.

[41] Chen R.B., Zhang K., Zhang H., Gao C.Y., Li C.L. (2018). Analysis of the antimicrobial mechanism of porcine beta defensin 2 against *E. coli* by electron microscopy and differentially expressed genes. Sci. Rep..

[42] do Nascimento V.V., Mello É.D.O., Carvalho L.P., de Melo E.J., Carvalho A.D.O., Fernandes K.V., Gomes V.M. (2015). PvD1 defensin, a plant antimicrobial peptide with inhibitory activity against Leishmania amazonensis. Biosci. Rep..

[43] Moretta A., Salvia R., Scieuzo C., Di Somma A., Vogel H., Pucci P., Sgambato A., Wolff M., Falabella P. (2020). A bioinformatic study of antimicrobial peptides identified in the Black Soldier Fly (BSF) *Hermetia illucens* (Diptera: Stratiomyidae). Sci. Rep..

[44] Shafee T.M., Lay F.T., Phan T.K., Anderson M.A., Hulett M.D. (2017). Convergent evolution of defensin sequence, structure and function. Cell. Mol. Life Sci..

[45] Pettersen E.F., Goddard T.D., Huang C.C., Couch G.S., Greenblatt D.M., Meng E.C., Ferrin T.E. (2004). UCSF Chimera—A visualization system for exploratory research and analysis. J. Comput. Chem..

[46] Di Somma A., Canè C., Moretta A., Duilio A. (2021). Interaction of Temporin-L Analogues with the *E. coli* FtsZ Protein. Antibiotics.

[47] Moretta A., Scieuzo C., Petrone A.M., Salvia R., Manniello M.D., Franco A., Lucchetti D., Vassallo A., Vogel H., Sgambato A. (2021). Antimicrobial Peptides: A New Hope in Biomedical and Pharmaceutical Fields. Front. Cell Infect. Microbiol..

[48] Tonk M., Vilcinskas A. (2017). The medical potential of antimicrobial peptides from insects. Curr. Top. Med. Chem..

[49] Xia J., Ge C., Yao H. (2021). Antimicrobial Peptides from Black Soldier Fly (*Hermetia illucens*) as Potential Antimicrobial Factors Representing an Alternative to Antibiotics in Livestock Farming. Animals.

[50] Parachin N.S., Mulder K.C., Viana A.A.B., Dias S.C., Franco O.L. (2012). Expression systems for heterologous production of antimicrobial peptides. Peptides.

[51] Yu H., Li H., Gao D., Gao C., Qi Q. (2015). Secretory production of antimicrobial peptides in *Escherichia coli* using the catalytic domain of a cellulase as fusion partner. J. Biotechnol..

[52] Li Y. (2011). Recombinant production of antimicrobial peptides in *Escherichia coli*: A review. Protein Expr. Purif..

[53] Harper S., Speicher D.W. (2011). Purification of proteins fused to glutathione S-transferase. Protein Chromatography.

[54] Zhao J., Li C., Chen A., Li L., Su X., Li T. (2010). Molecular characterization of a novel big defensin from clam *Venerupis philippinarum*. PLoS ONE.

[55] Vogel H., Müller A., Heckel D.G., Gutzeit H., Vilcinskas A. (2018). Nutritional immunology: Diversification and diet-dependent expression of antimicrobial peptides in the black soldier fly *Hermetia illucens*. Dev. Comp. Immunol..

[56] Roy A., Kucukural A., Zhang Y. (2010). I-TASSER: A unified platform for automated protein structure and function prediction. Nat. Protoc..

[57] Yang J., Yan R., Roy A., Xu D., Poisson J., Zhang Y. (2015). The I-TASSER Suite: Protein structure and function prediction. Nat. Methods.

[58] Yang J., Zhang Y. (2015). I-TASSER server: New development for protein structure and function predictions. Nucleic Acids Res..

[59] Ceroni A., Passerini A., Vullo A., Frasconi P. (2006). DISULFIND: A disulfide bonding state and cysteine connectivity prediction server. Nucleic Acids Res..

[60] Vullo A., Frasconi P. (2004). Disulfide connectivity prediction using recursive neural networks and evolutionary information. Bioinformatics.

[61] Frasconi P., Passerini A., Vullo A. A two-stage SVM architecture for predicting the disulfide bonding state of cysteines. Proceedings of the 12th IEEE Workshop on Neural Networks for Signal Processing.

[62] Ceroni A., Frasconi P., Passerini A., Vullo A. (2003). Predicting the disulfide bonding state of cysteines with combinations of kernel machines. J. Vlsi Signal Process. Syst. Signal Image Video Technol..

[63] Halder S., Yadav K.K., Sarkar R., Mukherjee S., Saha P., Haldar S., Sanmoy K., Sen T. (2015). Alteration of Zeta potential and membrane permeability in bacteria: A study with cationic agents. SpringerPlus.

