# Supplementary material for: Structural and Functional Characterization of a Novel Recombinant Antimicrobial Peptide from Hermetia illucens
Source: Curr Issues Mol Biol. 2021 Dec 21;44(1):1–13. doi: 10.3390/cimb44010001 (PMC8929087; doi:10.3390/cimb44010001)
Supplement: Supplementary file 1 [file cimb-44-00001-s001.zip › cimb-1450673-supplementary.pdf]

Structural and functional characterization of a novel recombinant Antimicrobial Peptide from *Hermetia illucens*

Angela Di Somma[1,2#], Antonio Moretta[3#], Carolina Cané[1], Carmen Scieuzo[3,4], Rosanna Salvia[3,4], Patrizia Falabella[3,4,*] and Angela Duilio[1,5*].

Supplementary Figure S1.

```
Hermetia     VTCDLLK----P-FFGRAPCMMHCILRFKKRTGFCSRQNVCVCR-     39
Copris       VTCDVLSFEAKGIAVNHSACALHCIAL-RKKGGSCQN-GVCVCRN     43
DLP4         ATCDLLS----PFKVGHAACAAHCIAR-GKRGGWCDKRAVCNCRK     40
AedesB       ATCDLLS----GFGVGDSACAAHCIAR-GNRGGYCNSQKVCVCRN     40
AedesA       ATCDLLS----GFGVGDSACAAHCIAR-GNRGGYCNSKKVCVCRN     40
             .***:*.        .. : *  ***    :: * *.   ** **
```

Supplementary Figure S1. Sequence homology of antimicrobial peptides C15867 to currently known AMPs.

Supplementary Figure S2.

(a)

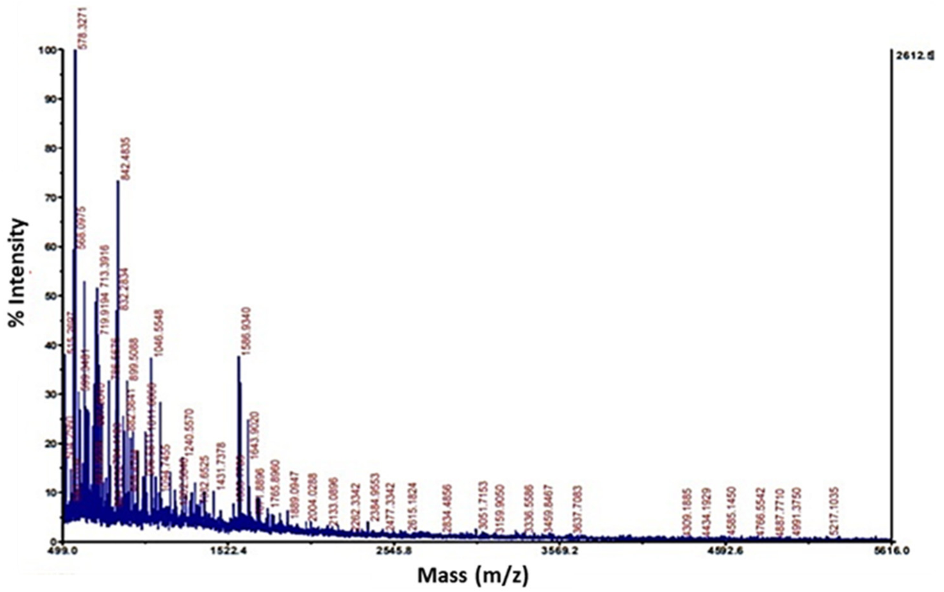

(b)

MSPILGYWKI KGLVQPTRLL LEYLEEKYEE HLYERDEGDK WRNKKFELGL EFPNLPYYID GDVKLTQSMA
IIRYIADKHN MLGGCPKERA EISMLEGAVL DIRYGVSRIA YSKDFETLKV DFLSKLPEML KMFEDRLCHK
TYLNGDHVTH PDFMLYDALD VVLYMDPMCL DAFPKLVCFK KRIEAIPQID KYLKSSKYIA WPLQGWQATF
GGGDHPPKSD **LVPRGSATCD LLSPFKVGHA ACALHCIAMG RRGGWCDGRA VCNCRREF**

Supplementary Figure S2. (a) MALDI-MS analysis of the tryptic digest of C15867 peptide. **(b)** Sequence coverage of the C15867 amino acid sequence. The peptides identified in the spectrum are underlined.

Supplementary Figure S3.

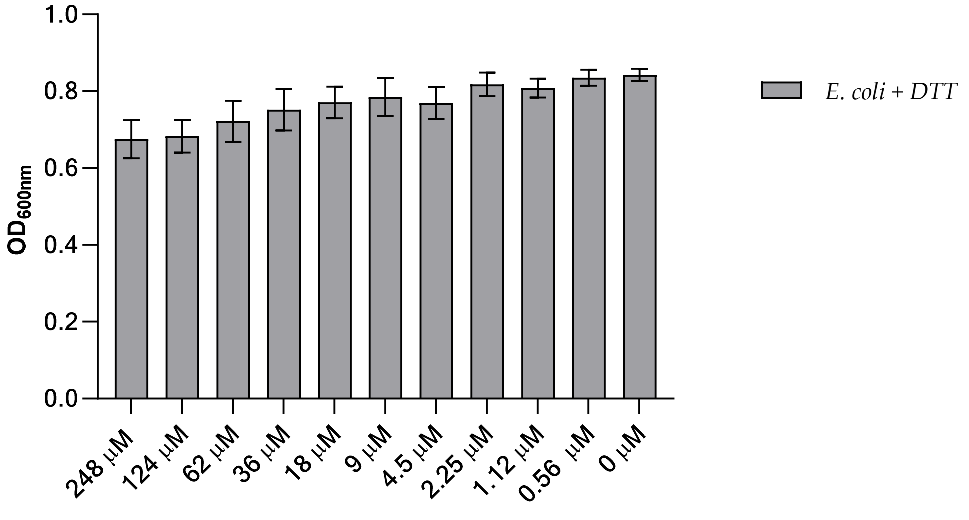

Supplementary Figure S3. Antimicrobial activity of the C-15867 peptide on *E.coli* cells following DTT treatment. *E.coli* cell growth was measured in the presence of different concentrations of the reduced C-158676 peptide. Bacterial cell growth was unaffected by the peptide in the reduced form up to 248 µM, showing that reduction of S-S bonds completely abolishes its antimicrobial activity.
